# Demographic and cardiovascular risk factors associated with blood flow characteristics of the left atrium and left atrial appendage

**DOI:** 10.1007/s00330-025-11866-w

**Published:** 2025-10-30

**Authors:** Maurice Pradella, Justin J. Baraboo, Stanley H. Chu, Anthony Maroun, Julia M. Hwang, Mitchell A. Collins, Amanda L. DiCarlo, Elizabeth K. Weiss, Lihui Zhao, Rod Passman, Susan R. Heckbert, Philip Greenland, Michael Markl

**Affiliations:** 1 Department of Radiology, Northwestern University Feinberg School of Medicine, Chicago, IL USA; 2 Department of Radiology, University Hospital Basel and University of Basel, Basel, Switzerland; 3 Department of Preventive Medicine, Northwestern University Feinberg School of Medicine, Chicago, IL USA; 4 Division of Cardiology, Department of Medicine, Northwestern University Feinberg School of Medicine, Chicago, IL USA; 5 Department of Epidemiology, University of Washington, Seattle, WA USA

**Keywords:** MRI, 4D-flow, Cardiovascular risk factors, Hemodynamics, Left atrium

## Abstract

**Objectives:**

Impaired left atrium (LA) and left atrial appendage (LAA) hemodynamics are risk factors (RF) for atrial thrombus formation and stroke. They can be assessed in vivo using 4D-flow-MRI; however, most studies involve clinical patient samples. We aimed to investigate demographic and cardiovascular (CV)-RF associations with LA and LAA hemodynamics in an observational study sample.

**Material and methods:**

Participants from the multi-ethnic study of atherosclerosis from our institution were approached to undergo cardiac MRI, including 4D-flow-MRI. LA and LAA volume, blood stasis (%voxel with velocity < 0.1 m/s), and peak velocity (PV, top 5% of voxels) were calculated. CV-RF (demographics, history of atrial fibrillation (AF), body mass index (BMI), etc.) were available through study records and investigated in multivariable linear regression models.

**Results:**

One hundred fifty-eight participants were included (age: 72.8 ± 7.3 years, 53% female). Higher age and AF were associated with lower PV_LA_ (β_age_: −0.16, *p* < 0.01; β_AF_: −2.81, *p* < 0.05) and higher LA stasis (β_age_: 0.64, *p* < 0.001; β_AF_: 5.60, *p* < 0.05). On the other hand, diabetes and left ventricular ejection fraction were associated with higher PV_LA_ (β_diabetes_: 3.29, *p* < 0.01; β_LVEF_: 0.11, *p* < 0.05), and diabetes was also associated with lower LA stasis (β: −6.12, *p* < 0.05). PV_LAA_ was lower in Black participants (β: −2.64, *p* < 0.01) and AF (β: −4.33, *p* < 0.001). LAA stasis was lower in males (β: −5.36, *p* < 0.01), white participants (β: −3.69, *p* < 0.05), diabetes (β: −4.54, *p* < 0.01) and higher BMI (β: −0.42, *p* < 0.05) while higher LA volume (β: 0.12, *p* < 0.01) was associated with higher LAA stasis.

**Conclusion:**

We identified RF associated with impaired LA and LAA hemodynamics in an observational study cohort.

**Key Points:**

***Question*** *Are there associations of demographic and CV factors with impaired atrial hemodynamics from 4D-flow cardiac MRI*?

***Findings*** *4D-flow MRI identified, amongst others, higher age, race, history of AF, and BMI in a sub-cohort of the Multi-Ethnic Study of Atherosclerosis*.

***Clinical relevance*** *Understanding risk factor associations with atrial hemodynamics could aid in identifying subclinical atrial thrombus formation risks, potentially allowing for earlier preventive strategies against diseases such as stroke in diverse populations*.

**Graphical Abstract:**

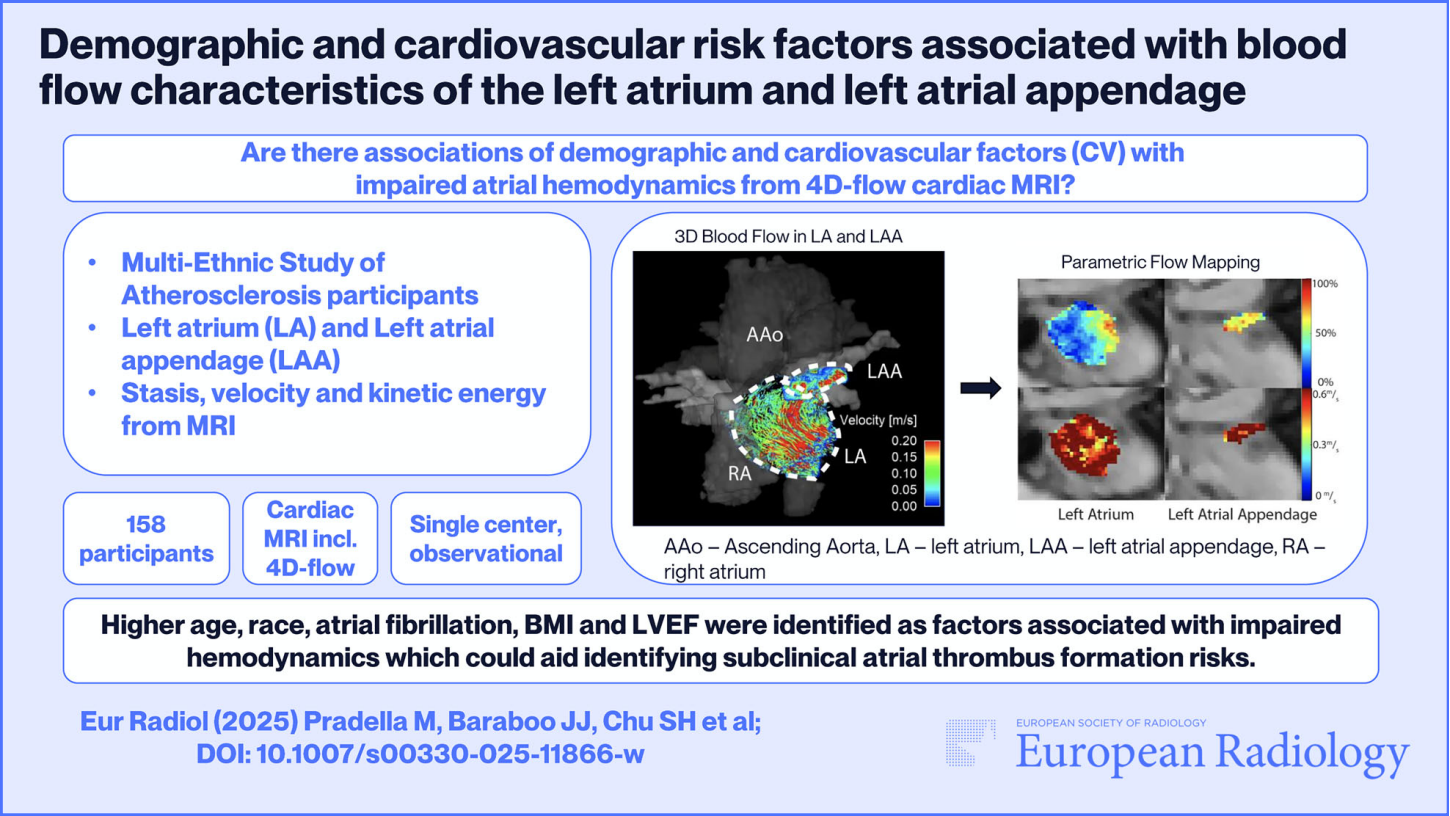

## Introduction

Left atrial enlargement was first associated with stroke and death in the Framingham Heart Study and has since been linked to increased cardiovascular (CV) risk [1]. Besides volume, multiple other left atrial parameters were established for assessment of left atrium (LA) function, for example, emptying fraction or strain [2, 3]. Additional characterization of the LA is now available using four-dimensional (4D) flow cardiac MRI. This technique enables 3D assessment of blood flow dynamics in vivo with full volumetric coverage of the entire LA and left atrial appendage (LAA), which allows investigation of atrial hemodynamic parameters such as blood stasis and peak velocity [4, 5]. Shen et al reported the potential to identify atrial myopathy based on LA and LAA flow characteristics. This is of interest since there is a potential association between thrombus formation in the LA and/or LAA with the development of stroke as a consequence of impaired atrial blood flow (increased flow stasis, reduced peak velocities) [6]. Therefore, blood flow assessment might help guide the treatment of stroke prevention in atrial fibrillation (AF) [7]. However, there is a lack of knowledge of the associations of atrial 4D-flow parameters with demographic and CV risk factors in general population studies. This is ascribed to LA flow assessment being typically performed in patient cohorts, mainly in those with known AF [8, 9].

The multi-ethnic study of atherosclerosis (MESA) previously contributed to better understanding the role of the LA using cardiac MRI, for example, in regard to demographic and CV risk factors, incident AF, or incident cerebrovascular events [10–12].

The aim of this study was to investigate associations of demographic and CV risk factors with LA and LAA flow characteristics derived from 4D-flow MRI in MESA participants at a single MESA study site.

## Methods

### Study population

MESA is an ongoing study to investigate the development and progression of subclinical CV disease [13]. The current study is a cross-sectional sub-study conducted at MESA examination 6, with an additional cardiac MRI performed later; it was approved by the institutional review board. The recruitment approach for MESA at Northwestern University included Chinese, Black, and White participants. Participants were free of clinical CV disease at baseline and have been followed over more than two decades, including a total of six detailed examinations. MESA participants at the Chicago, IL site were approached to undergo an additional cardiac MRI, including a 4D-flow series (2018–2020). Inclusion criteria were previous enrollment and ongoing participation in MESA. There were no formal exclusion criteria.

Demographic data and information on CV risk factors were available through the MESA examination 6 (between 2016 and 2018); the process of obtaining those was previously described [14]. Demographic and CV risk factors investigated were age, sex, race, body mass index (BMI), diabetes, arterial hypertension, history of AF, current smoker (vs former/never smoker), present alcohol consumption, and impaired left ventricular (LV) function. A seated blood pressure test was performed to assess hypertension, defined as systolic blood pressure ≥ 140 mmHg and/or diastolic blood pressure ≥ 90 mmHg. In addition, participants were asked during an interviewer-administered questionnaire if they were ever told by a doctor that they had hypertension or diabetes and/or take specific medication. History of AF was defined in four different ways according to the ancillary study of AF in MESA [15]: (1) identification of AF on study ECG (electrocardiogram, which were read at a centralized ECG reading center (Epidemiological Cardiology Research Center, Wake Forest University)), (2) identification of AF during 14-day ECGs with a minimum duration of 30 s, (3) hospital discharge diagnosis of AF according to International Classification of Diseases, and (4) participants enrolled in Medicare with in- or outpatient AF claims. Because of the importance of AF history in regard to LA function, all participants had undergone two or three 14-day ECG monitors (ZioPatch, iRhythm) in order to detect additional subclinical AF.

### Cardiac MRI and 4D-flow

Cardiac MRI was performed on a 1.5-T MRI scanner (Aera, Siemens HealthCare). All participants underwent standard-of-care MRI, including retrospective ECG-gated time-resolved (CINE) balanced steady-state free precession imaging in short-axis orientation of the LV to assess left ventricular ejection fraction (LVEF).

Furthermore, retrospective ECG-gated time-resolved 3D phase-contrast (PC) MRI with 3D velocity encoding (4D-flow) MRI was acquired in coronal orientation during free breathing. 4D-flow parameters: TE = 2.44 ms, TR = 5.16 ms, flip angle = 7°, resolution = 2.5 × 2.5 × 2.5 mm^3^, FOV = 480 × 400, matrix = 192 × 120, number of cardiac time frames = 22, VENC = 120 cm/s, compressed sensing with *R* = 10. Of note, participants did not receive contrast media.

### 4D-flow analysis

**Fig. 1:**
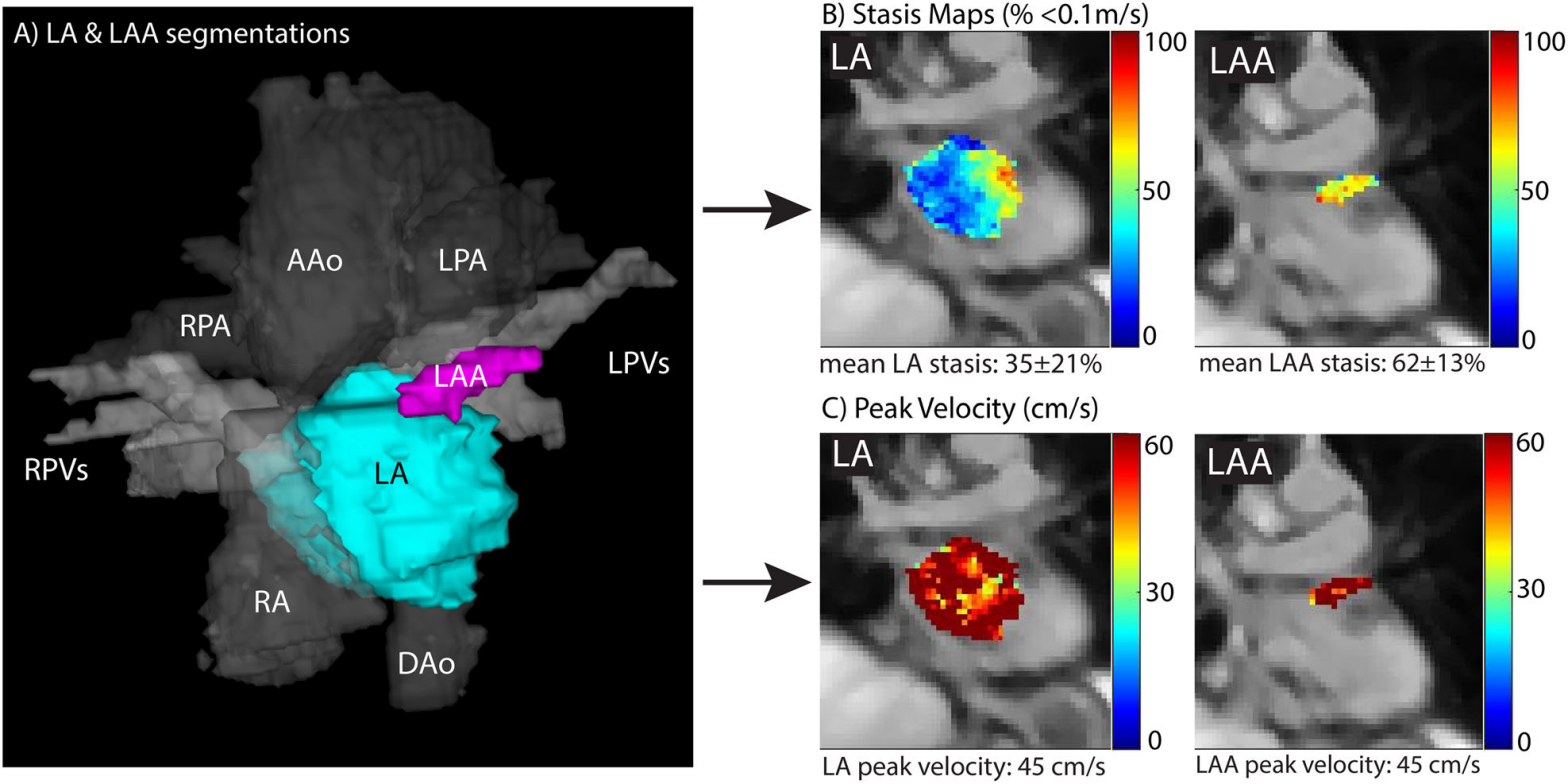
Analysis workflow. **A** LA and LAA were segmented on 3D Phase Contrast MRA images for each participant, and velocities were masked. Based on these segmentations, stasis (**B**) and peak velocity (**C**) were calculated, and respective MIP images were created. AAo, ascending aorta; DAo, descending aorta; LA, left atrium; LAA, left atrial appendage; LPA, left pulmonary artery; LPV, left pulmonary vein; RA, right atrium; RPA, right pulmonary artery; RPV, right pulmonary vein

Data analysis was described in detail elsewhere [4]. Briefly, 4D flow MRI data were corrected for Maxwell terms and eddy currents. LA and LAA were segmented on 3D PC-MR angiography series by a CV radiologist with 3 years’ experience (Mimics, Materialize), Fig. 1A. The respective boundaries of the LA and LAA were contoured on each slice where they were visible, and the resulting LA and LAA segmentations were used to mask the 4D flow data in order to calculate atrial volumes and derive maps of blood stasis and peak velocity for the LA and LAA (Fig. 1B, C). Blood stasis was calculated as the relative number of imaging voxels inside the LA and LAA segmentations with < 10 cm/s per time point, normalized by the total number of time points. Peak velocity was calculated as the top 5% of voxels per cardiac time point. Kinetic energy was calculated at each cardiac time point for each voxel using Eq. 1, assuming a constant density of blood (ρ) of 1060 kg/m^3^. Kinetic energies were then summed over the cardiac cycle and averaged over the LA and LAA volumes.

1 $${KE}=\frac{1}{2}\rho * {voxel\; volume}* {({{velocity\; magnitude}})}^{2}$$

### Intra- and inter-reader assessments

The CV radiologist performed re-segmentation on randomly selected 20 cases, blinded to the initial results, to assess intra-reader reproducibility after several months. For inter-reader assessment, a postdoctoral fellow (A.L.D.) with 4 years’ experience in cardiac MRI segmented LA and LAA in the same fashion.

### Statistical methods

Statistical analysis was performed in Python (Python Software Foundation). 4D-flow parameters (peak velocity and stasis for LA and LAA) were compared to continuous parameters using the Pearson r correlation. Comparison of binary discrete parameters was performed (after testing for normal distribution (Kolmogorov–Smirnov test)) using either Student’s *t*-test (normal distribution) or Mann–Whitney *U*-test (non-normal distribution). Associations with race were evaluated using either one-way ANOVA or the Kruskal–Wallis test and followed by post hoc analysis. A *p* value < 0.05 was considered significant.

Inter-reader and intra-reader comparisons were performed using intra-class correlations (ICC) for volume, stasis, peak velocity, and kinetic energy of the LA and LAA.

For LA stasis, LA kinetic energy, LA peak velocity, LAA stasis, LAA kinetic energy, and LAA peak velocity, multivariable stepwise linear regression models were performed to assess the associations of demographic and CV risk factors. Race (White, Black, or Chinese) was entered as a separate variable. In addition, because of unexpected observations related to diabetes, we performed subgroup analysis in participants with and without diabetes. Lastly, multivariable linear regression models including all demographic and CV risk factors were calculated.

## Results

### Study sample

**Fig. 2:**
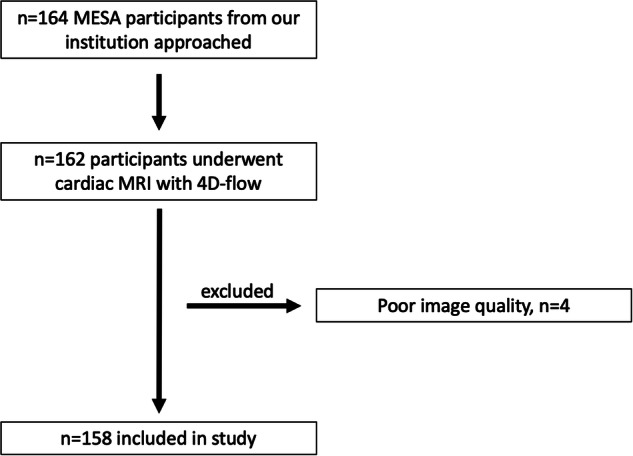
Flowchart of participants included in this study

Demographics, CV risk factors, and LV parameters can be found in Table 1. One hundred sixty-four participants were initially approached, of which 162 underwent cardiac MRI with 4D-flow (Fig. 2). Four participants had to be excluded due to poor image quality due to image noise. Finally, a total of 158 participants were included in this study (Mean age: 72.8 ± 7.3 years, 53% female). Race was self-reported as Black (23%), Chinese (33%), or White (44%). Hypertension was present in 57%, diabetes mellitus in 13% and a history of AF in 12% of the entire sample.

**Table 1:** Characteristics of study cohort

Parameter	
Number of participants	158
Age, years	72.8 ± 7.3
Sex, female	83 (52.5%)
Race	
Black	36 (22.8%)
Chinese	52 (32.9%)
White	70 (44.3%)
BMI, kg/m^2^	26.4 ± 4.7
CV risk factors	
Diabetes	20 (12.7%)
Hypertension	90 (57.0%)
History of AF	19 (12.0%)
Present alcohol consumption	75 (47.5%)
Current smoker	5 (3.2%)

Characteristics of participants included in this study

### Demographics associated with flow parameters

**Fig. 3:**
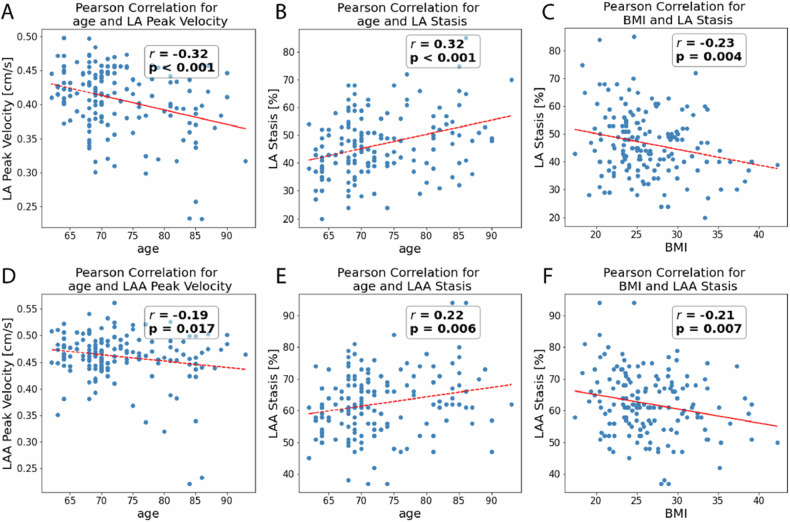
Correlation analyses of continuous parameters. This figure shows significant correlations of age and BMI with stasis and peak velocity in the LA (**A**–**C**) and LAA (**D**–**F**). BMI, body mass index; LA, left atrium; LAA, left atrial appendage

We found statistically significant correlations between demographics and multiple 4D-flow parameters: Increased age showed moderate but significant correlation with increased LA stasis (*r* = 0.32, *p* < 0.01), reduced LA peak velocity (*r* = −0.32, *p* < 0.01), reduced LA kinetic energy (*r* = −0.35, *p* < 0.01), increased LAA stasis (*r* = 0.22, *p* < 0.01), reduced LAA peak velocity (*r* = −0.19, *p* < 0.02) and LAA kinetic energy (*r* = −0.17, *p* = 0.03); Table 2 and Fig. 3A, B, D, E. Female participants had higher stasis in the LAA (*p* < 0.01). Furthermore, LAA peak velocity was significantly different between races (*p* = 0.049); Table 3. Post hoc test attributed this result to White participants having significantly higher LAA peak velocities than Black participants (*p* = 0.044). LAA kinetic energy was also significantly different between races (*p* < 0.01), with Black participants having lower LAA kinetic energy compared to both White and Chinese participants (*p* < 0.01 and *p* = 0.02, respectively).

**Table 2:** Result of correlation analyses

Parameter	LA peak velocity	LA stasis	LA kinetic energy	LAA peak velocity	LAA stasis	LAA kinetic energy
	Pearson *r*	Pearson *r*	Pearson *r*	Pearson *r*	Pearson *r*	Pearson *r*
age	**−0.32 (*p*** < **0.001)**	**0.32 (*p*** < **0.001)**	**−0.35 (*p*** < **0.001)**	**−0.19 (*p*** = **0.017)**	**0.22 (*p*** = **0.006)**	**−0.17 (*p*** = **0.03)**
BMI	0.15 (*p* = 0.053)	**−0.23 (*p*** = **0.004)**	0.11 (*p* = 0.17)	0.13 (*p* = 0.11)	**−0.21 (*p*** = **0.007)**	0.15 (*p* = 0.07)

Correlations of age and BMI with peak velocity, stasis, and kinetic energy in the LA and LAA. Significant results are in bold

*BMI* body mass index, *LA* left atrium, *LAA* left atrial appendage

**Table 3:** Univariable comparisons

Parameter	LA peak velocity [m/s]	LA stasis [%]	LA mean kinetic energy [10^−9^*J*]	LAA peak velocity [m/s]	LAA stasis [%]	LAA mean kinetic energy [10^−9^*J*]
Sex (female/male)	0.40 ± 0.05/0.41 ± 0.04	48.0 ± 12.2/44.9 ± 10.7	12.8 ± 4.8/13.5 ± 4.1	0.46 ± 0.05/0.47 ± 0.04	**64.0** ± **12.0/61.0** ± **11.5**	21.2 ± 9.8/21.6 ± 1.1
*p* value	0.08	0.09	0.30	0.11	**0.005**	0.83
Race (Black/Chinese/White)	0.4 ± 0.06/0.42 ± 0.05/0.42 ± 0.06	45.5 ± 18.8/45.0 ± 13.5/45.5 ± 13.8	11.1 ± 4.8/14.3 ± 6.1/12.1 ± 6.3	**0.45** ± **0.05/0.46** ± **0.03/0.47** ± **0.04**	66.5 ± 13.2/61.0 ± 7.2/61.5 ± 14.8	**14.9** ± **1.2/21.2** ± **8.4/21.3** ± **1.2**
*p* value	0.23	0.82	0.06	**0.049**	0.23	**0.007**
Diabetes (no/yes)	**0.41** ± **0.06/0.45** ± **0.03**	**47.5** ± **15.0/40.0** ± **8.8**	**12.1** ± **6.0/15.7** ± **3.5**	0.46 ± 0.05/0.47 ± 0.04	**63.0** ± **9.6/57.0** ± **10.0**	**20.5** ± **9.1/26.1** ± **1.2**
*p* value	**0.001**	**0.001**	**<** **0.001**	0.187	**0.01**	**0.015**
Hypertension (no/yes)	0.41 ± 0.04/0.4 ± 0.05	45.7 ± 10.0/47.2 ± 12.7	12.9 ± 4.9/13.4 ± 3.9	**0.47** ± **0.03/0.45** ± **0.05**	**60.2** ± **8.7/63.6** ± **10.5**	20.4 ± 1.1/22.7 ± 9.5
*p* value	0.21	0.42	0.42	**0.048**	**0.03**	0.15
History of AF (no/yes)	**0.41** ± **0.04/0.37** ± **0.07**	**44.0** ± **13.8/51.0** ± **23.0**	**13.5** ± **4.3/11.7** ± **4.9**	0.47 ± 0.04/0.46 ± 0.1	**61.6** ± **8.9/66.6** ± **15.0**	20.1 ± 1.1/19.7 ± 1.9
*p* value	**<** **0.001**	**0.008**	**0.009**	0.1	**0.036**	0.22
Alcohol consumption (no/yes)	0.4 ± 0.05/0.41 ± 0.05	46.6 ± 12.1/46.5 ± 11.1	12.8 ± 4.5/13.5 ± 4.4	0.46 ± 0.04/0.46 ± 0.05	61.6 ± 10.5/62.7 ± 9.2	21.3 ± 1.1/22.2 ± 9.5
*p* value	0.38	0.96	0.34	0.93	0.48	0.92
Current/former smoker (no/yes)	0.41 ± 0.05/0.41 ± 0.05	46.2 ± 12.2/46.9 ± 11.1	13.3 ± 4.9/13.0 ± 4.1	0.46 ± 0.05/0.46 ± 0.04	61.7 ± 10.5/62.6 ± 9.3	21.2 ± 1.0/21.5 ± 1.0
*p* value	0.7	0.7	0.65	0.63	0.59	0.84

Results of univariable comparisons between demographic and CV risk factors for peak velocity, stasis, and kinetic energy in both LA and LAA. Significant results are in bold

*AF* atrial fibrillation, *LA* left atrium, *LAA* left atrial appendage

### CV risk factors associated with flow parameters

Lower stasis in both LA and LAA was associated with higher BMI (*r* = −0.23, *p* < 0.01 and *r* = −0.21, *p* < 0.01, respectively) and diabetes (each *p* < 0.01, respectively); Tables 2 and 3. Figure 3C, F. Conversely, higher stasis in the LA and LAA was associated with AF history (*p* < 0.01 and *p* < 0.036, respectively) while higher LAA stasis alone was associated with hypertension (*p* = 0.033).

Increased LA peak velocity was associated with diabetes (*p* < 0.01), while lower LA peak velocity showed a significant association with AF history (*p* < 0.01). Lower LAA peak velocity was associated with hypertension (*p* = 0.048).

Lastly, increased kinetic energy in both LA and LAA was associated with diabetes (*p* < 0.01, *p* < 0.02; respectively), while lower LA kinetic energy was also associated with AF history (*p* < 0.01).

### Multivariable models to identify predictors of atrial hemodynamics

#### Peak velocity

**Fig. 4:**
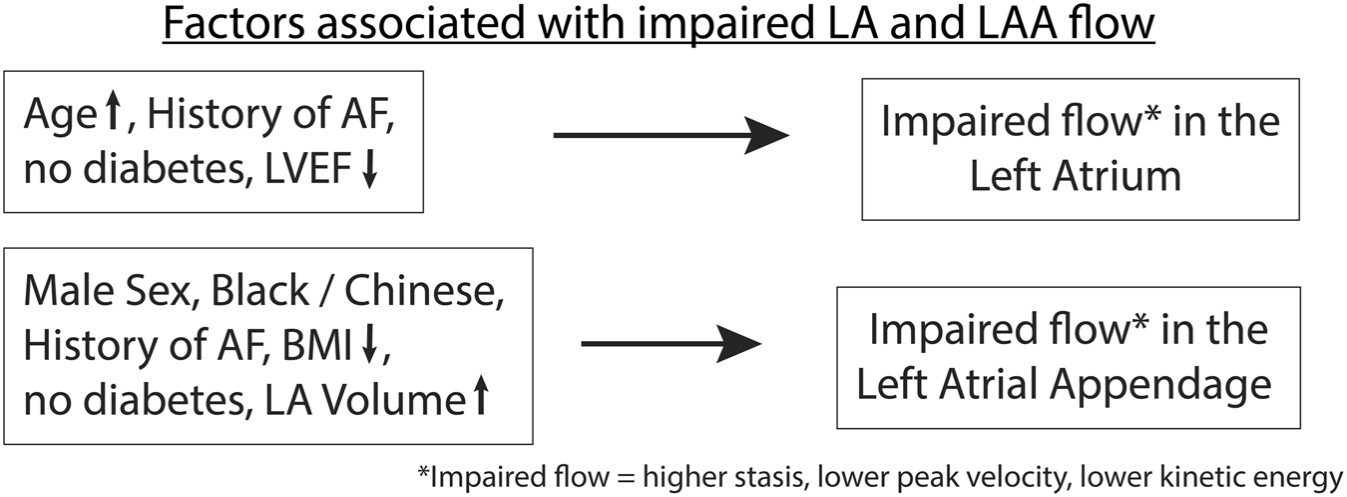
Results from multivariable analysis. In stepwise linear multivariable analysis, we were able to identify risk factors associated with impaired flow (higher stasis, lower peak velocity) for both the LA and LA appendage; those could indicate an increased risk for thrombus formation

Factors that were associated with a higher LA peak velocity were younger age (β: −1.65 [per 10-years], *p* < 0.01), diabetes (β: 3.29, *p* < 0.01), and higher LVEF (β: 0.11, *p* = 0.03) while history of AF (β: −2.81, *p* = 0.013) was associated with lower peak velocity; Table 4 (Model 1). For the LAA, Black race (β: −2.64, *p* < 0.01) and history of AF (β: −4.33, *p* < 0.01) were predictors of lower peak velocity; Table 4 (Model 4) and Fig. 4.

**Table 4:** Multivariable models

	Model 1: LA peak velocity	Model 2: LA Stasis	Model 3: LA mean kinetic energy	Model 4: LAA peak velocity	Model 5: LAA stasis	Model 6: LAA mean kinetic energy
Model *R*^2^	0.209	0.184	0.197	0.151	0.188	0.156
Model *p* value	< 0.001	< 0.001	< 0.001	< 0.001	< 0.001	< 0.001
Parameters						
Age, per 10 years	−1.65**	4.04***	−1.94***			
Male sex					−5.36**	
Race						
Black				−2.64**		−5.77*
White					−3.69*	
History of AF	−2.81*	6.69*		−4.33***		
Diabetes	3.29**	−6.92**	3.50***		−4.55*	4.70*
LVEF, per %	0.11*		0.10*			
LA volume, per mL					0.12**	−0.10**
BMI, per kg/m^2^					−0.42*	0.41*

This table displays the multivariable stepwise linear regression models for LA peak velocity, LA stasis, LA kinetic energy, LAA peak velocity, LAA stasis, and LAA kinetic energy. Covariates that were significantly associated with each LA or LAA variable are shown. Covariates that were not associated with 4D-flow parameters in any multivariable model were: Chinese race, hypertension, smoking, and alcohol consumption

*AF* atrial fibrillation, *BMI* body mass index, *LA* left atrium, *LAA* left atrial appendage, *LVEF* left ventricular ejection fraction

* *p* < 0.05, ** *p* < 0.01, *** *p* < 0.001

#### Stasis

Factors associated with higher stasis in the LA were increased age (β: 4.04, *p* < 0.01), and history of AF (β: 6.69, *p* = 0.037), while diabetes was associated with lower stasis (β: -6.92, *p* < 0.02); Table 4 (Model 2). In regard to LAA stasis, higher LA volume (β: 0.12, *p* < 0.01) was associated with increased stasis. White participants (β: −3.69, *p* < 0.02), male sex (β: −5.36, *p* < 0.01), a lower BMI (β: −0.42, *p* < 0.01), and diabetes (β: −4.55, *p* = 0.04) were independent predictors of lower LAA stasis; Table 4 (Model 5) and Fig. 4.

#### Kinetic energy

In the model for the LA, increased mean kinetic energy was associated with younger age (β: −1.94, *p* < 0.01), presence of diabetes (β: 3.50, *p* < 0.01), and higher LVEF (β: 0.10, *p* = 0.048); Table 3 (Model 3). For the LAA, Black race (β: −5.77, *p* = 0.046) and smaller LA volume (β: −0.10, *p* < 0.01) were associated with lower kinetic energy while diabetes (β: 4.70, *p* < 0.049) and higher BMI (β: 0.41, *p* < 0.047) were associated with higher mean kinetic energy.

Multivariable models for peak velocity, stasis, and kinetic energy in LA and LAA, including all covariates, can be found in the supplement. Participants with and without diabetes.

Since we observed lower stasis, higher peak velocities and also higher kinetic energy in participants with diabetes, we investigated baseline parameters (age, sex, race, BMI, LA volume, LVEF and other CV risk factors) in those subgroups further: BMI was significantly higher in participants with diabetes (no diabetes: 26.0 ± 4.4 kg/m^2^, diabetes: 28.2 ± 6.2 kg/m^2^, *p* = 0.049). Furthermore, participants without diabetes had higher LVEF (67.9 ± 8.8% vs 60.5 ± 7.4%, *p* < 0.01). All other parameters were not significantly different (Table 5).

**Table 5:** Subgroup analysis for diabetes

Parameter	No diabetes (*n* = 136)	Diabetes (*n* = 20)	*p* value
Age, years	73.0 ± 7.5	70.0 ± 4.9	0.09
Sex, female	74 (54.4%)	7 (35.0%)	0.17
Race			0.37
Black	64 (47.1%)	6 (30.0%)	
Chinese	43 (31.6%)	9 (45.0%)	
White	29 (21.3%)	5 (25.0%)	
BMI, kg/m^2^	26.0 ± 4.4	28.2 ± 6.2	**0.049**
LA Volume, mL	67.4 ± 20.2	66.0 ± 18.1	0.77
LVEF, %	67.9 ± 8.8	60.5 ± 7.4	**<** **0.001**
CV risk factors			
Hypertension	76 (55.9%)	7 (35.0%)	0.60
History of AF	11 (8.1%)	1 (5.0%)	0.93
Present alcohol consumption	75 (55.1%)	8 (40.0%)	0.30
Current smoker	5 (3.7%)	0 (0%)	0.68

Displays a comparison of demographic and CV risk factors of participants with and without diabetes. We found significantly lower LVEF and a slightly higher BMI in participants with BMI (marked in bold), all other parameters did not differ significantly

*BMI* body mass index, *LA* left atrial, *LVEF* left ventricular ejection fraction

### Intra- and inter-reader assessments

ICC showed excellent agreement between the two readers for all LA parameters: highest agreement was seen for LA stasis (0.99), LA peak velocity (0.98), LA kinetic energy (0.97), and LA volume (0.95). Results for LAA parameters were moderate to excellent: LAA peak velocity (0.94), LAA stasis (0.93), LAA kinetic energy (0.92), and LAA volume (0.76). ICC for intra-reader assessment revealed high agreement for LA parameters (LA stasis (0.91), LA peak velocity (0.83), LA volume (0.88) and LA kinetic energy (0.73)) and moderate agreement for LAA parameters (LA stasis (0.55), LA peak velocity (0.66), LA volume (0.62), and LA kinetic energy (0.44)).

## Discussion

In this study, we investigated associations of demographic and CV risk factors with blood flow dynamics in the LA and LAA in a sample of participants who were drawn 18 years earlier from the general population, free of clinically recognized CV disease at initial examination. We found that the history of AF and diabetes were the only independent parameters associated with both LA and LAA flow hemodynamics in multivariable models. While AF resulted in impaired blood flow, diabetes was associated with higher LA peak velocity and lower stasis in both LA and LAA. Furthermore, older age and impaired LV function were each associated with impaired LA flow (higher LA stasis, lower LA peak velocity) while female sex, Black race, lower BMI, normal glucose status (vs diabetes), and larger LA volume were associated with impaired LAA flow parameters (higher LAA stasis, lower LAA peak velocity).

### 4D-flow MRI of the LA

Blood flow dynamics in the LA and LAA have been investigated for several years, however, interest has increased in recent years because of the potential association between thrombus formation in the LA and LAA leading to stroke [7, 16–18]. Flow hemodynamics are typically evaluated using echocardiography, which is widely available and offers assessment of velocities at certain planes, for example, LAA emptying or at the mitral valve. Bernhardt et al reported significantly lower LAA peak velocity in patients with stroke [19]. 4D-flow MRI, on the other hand, enables one to perform 3D-based flow assessment, which captures the entire LA and/or LAA [4]. This allows measurement of velocities globally in the respective volumes, resulting in advanced parameters such as blood flow stasis for the LA and LAA. However, these techniques have so far been mainly applied to patient cohorts with AF; studies in unselected individuals are limited [8, 20]. In inter-reader assessment, we found high agreement for stasis, peak velocity, kinetic energy, and volumes for the LA. While LAA volume only showed moderate agreement, LAA stasis, kinetic energy, and peak velocity also showed excellent agreement. On the other hand, intra-reader assessment only revealed moderate agreement for the LA and LAA parameters. In the future, automatic segmentation of the LA and LAA using advanced techniques such as deep learning might help to increase robustness even further [21, 22].

### CV risk factors and hemodynamics

In our study, in MESA participants from our institution, we were able to identify CV risk factors associated with blood flow dynamics. Most importantly, a history of AF was associated with impaired flow parameters in the LA and LAA significantly in univariate analyses, which was confirmed by multivariable models. We thereby aimed to detect silent AF by applying up to three 14-day monitors in each participant. AF history was independently associated with LA and LAA peak velocities, as well as LA stasis, which is in accordance with a report on healthy controls in a study on AF patients [20]. Furthermore, AF history was not associated with LAA stasis in multivariable models, similar to a previous study which included 30 AF patients and 15 controls [20]. This is of interest since the LAA is the primary location for thrombi found in the left heart, which are linked to stroke [23]. Shen et al suggested that increased LAA stasis in atrial myopathy is, also in the absence of AF, associated with thrombus formation [7]. However, one must consider that finding > 90% of thrombi in the LAA does not mean that they originated in the LAA. They could have originated elsewhere, for example, in LA, and then got dislodged into the LAA.

Diabetes was also associated with altered flow hemodynamics in the LA and LAA. Univariate analyses revealed lower stasis in the LA and LAA in participants with diabetes, and LA peak velocity was found to be higher. These observations were confirmed in multivariable models, which identified diabetes as significantly associated with higher LA peak velocity, as well as lower stasis in the LA and LAA. We did not anticipate finding a beneficial association of diabetes with LA and LAA blood flow when conducting this study. Compared to hypertension, which was homogeneously distributed in our cohort (90/158 participants had hypertension) and which was not associated with impaired flow in multivariable analyses, only 20/158 participants were diagnosed with diabetes. Diabetes could potentially induce atrial myopathy via inflammation and thereby contribute to thrombus formation [7, 24]. On the other hand, it is known that diabetes increases the risk of AF and stroke by up to 40% (our data showed the worsening effect of AF on flow hemodynamics, too), the potential mechanism by which diabetes is associated with greater LA blood flow dynamics is unclear and needs to be investigated in further studies [25]. Detailed analysis of participants with and without diabetes showed that participants with diabetes had higher BMI and lower LVEF. These differences might have biased the observed associations of diabetes with flow parameters. Moreover, a CMR study in patients with diabetes found higher peak velocity at the level of the mitral valve compared to normoglycemic controls using 2D-phase contrast imaging [26]. Diastolic dysfunction might contribute to this finding, but further studies are required to investigate this in more detail.

Higher BMI was associated with lower stasis in both LA and LAA. In AF patients, there is evidence of a possible effect of obesity on thrombus formation, which is thought to be related to higher stasis. However, this is controversial and data from the general population are limited [27, 28]. Our data suggests that there is no association or possibly a protective association of higher BMI with regard to LA and LA appendage blood flow. Smoking status or alcohol consumption as CV risk factors were not associated with blood flow dynamics in the LA and LAA in our cohort. Of note, our cohort only included a few active smokers, and alcohol consumption in MESA is generally low (less than three standard drinks per week) [29].

Very limited information is available from prior studies on the association of CV risk factors with measures of LA or LAA flow parameters. Similar to our finding, Agmon et al reported lower LAA velocities with age in a general population study using echocardiography [30]. Furthermore, they reported higher LAA velocities in men compared to women, which is partially in accordance with our finding that men had lower LAA stasis, however, peak velocity was not associated with sex. Zemrak et al reported on the association of race with atrial characteristics; they showed that Chinese Americans have smaller LA. In our analysis of flow characteristics, White participants had lower LAA stasis. LA size as an independent variable was only associated with LAA stasis but not with flow parameters of the LA [10]. Results from the stepwise and full regression models were largely consistent, supporting the robustness of the observed associations.

From a clinical perspective, anticoagulation is recommended for all AF patients with two or more vascular risk factors or patients with LAA thrombus to prevent stroke and other thromboembolic complications [31–34]. However, anticoagulation therapy also carries an increased risk of bleeding, including spontaneous hemorrhages such as intracranial bleeding, as well as a heightened risk of bleeding following trauma [33, 34]. The limitations of the discriminative value of the recommended risk stratification scoring system, CHADS2VASC2, are well appreciated. A more detailed understanding of the specific risk factors contributing to thrombus formation may aid in better identifying patients who are most likely to benefit from anticoagulation. Moreover, patients identified as high-risk who are poor candidates for chronic anticoagulation could be considered for alternative strategies, such as LAA closure procedures [35]. Nonetheless, further studies are needed to validate these approaches and guide clinical decision-making.

### Limitations

This study has several limitations. First, it is a single-center study. MRI exams were acquired on a scanner from one vendor. These limitations were mainly related to 4D-flow being a rather new technique that requires well-trained technologists, as well as advanced knowledge for complex data processing. Participants were part of the MESA study and, therefore, of older age and do not represent a cross-section of the general population; moreover, due to the impact of the COVID-19 pandemic at our institution, we were not able to recruit more participants for this study. The latter also limits our ability to add more variables to our multivariable analyses. Test–retest repeatability of 4D-flow measurements was not included in our study setting. Furthermore, we were only able to include White, Black, and Chinese participants. Other races/ethnicities were not recruited by our field center for this study. In addition, while MESA is a multi-ethnic study, our cohort might not be representative of the general population, which limits generalizability. Mitral valve regurgitation was not systematically assessed in our cohort, which could have affected peak velocity measurements in the LA. There was a time gap between MESA exam 6 (when the CV risk factors were reported) and the MRI exam, participants could have developed CV disease in between. In addition, there is currently no follow-up data on the study participants available; however, MESA exam 7, which will take place in 2024/2025, might enable future investigations focusing on events such as stroke or other major adverse events.

Moreover, the number of current smokers and participants with a high level of alcohol consumption was low, limiting study power. Up to three 14-day ECG monitors were performed in our participants to detect subclinical AF, which was found in 19 participants (12%) of our sample. However, rhythm during 4D-flow acquisition was not specifically monitored. Lastly, our results suggest that diabetes was associated with lower LA stasis, and higher BMI was associated with lower LAA stasis; while we mention potential hypotheses, these findings were unexpected and might be related to the small sample size.

In this study, we describe associations of demographics and CV risk factors with flow characteristics in the LA and LAA in a community-based multi-ethnic cohort of older individuals. In multivariable models, higher LAA stasis, a parameter thought to be linked to thrombus formation in the LAA, was associated with female sex, higher LA volume, lower BMI, race other than White, and absence of diabetes. Furthermore, a history of AF was associated with lower peak velocities in the LA and LAA, as well as higher LA stasis, while older age was linked to impaired flow in the LA. Diabetes, which was present in about 12% of participants, seemed to improve flow characteristics, which might be impacted by a rather small share of participants with diabetes. The overall impact of lower velocities and higher stasis parameters on thrombus formation and stroke needs to be investigated in further larger studies.

## Supplementary information

ELECTRONIC SUPPLEMENTARY MATERIAL

## Data Availability

The data sets used for the current study are available from the corresponding author on reasonable request.

## Abbreviations

4D: Four-dimensional
AF: Atrial fibrillation
BMI: Body mass index
CV: Cardiovascular
ECG: Electrocardiogram
LA: Left atrium
LAA: Left atrial appendage
LV: Left ventricular
LVEF: Left ventricular ejection fraction
MESA: Multi-ethnic study of atherosclerosis
