# Supplementary material for: Demographic and cardiovascular risk factors associated with blood flow characteristics of the left atrium and left atrial appendage
Source: Eur Radiol. 2025 Oct 30;36(4):3079–90. doi: 10.1007/s00330-025-11866-w (PMC13035651; doi:10.1007/s00330-025-11866-w)
Supplement: Supplementary file 1 — ELECTRONIC SUPPLEMENTARY MATERIAL [file 330_2025_11866_MOESM1_ESM.pdf]

Demographic and Cardiovascular Risk Factors associated with Blood Flow Characteristics of the Left Atrium and Left Atrial Appendage

ELECTRONIC SUPPLEMENTARY MATERIAL

Supplement Table 1 Multivariable models including all risk factors

	**Model 1: LA peak velocity**	**Model 2: LA stasis**	**Model 3: LA mean kinetic energy**	**Model 4: LAA peak velocity**	**Model 5: LAA stasis**	**Model 6: LAA mean kinetic energy**
Model R2	0.253	0.248	0.243	0.257	0.237	0.161
Model p value	<0.001	<0.001	<0.001	<0.001	<0.001	0.007
**Parameter**						
Age, per 10 years	-1.55**	3.52**	-1.85***	-0.48	1.36	-1.06
Male Sex	1.37	-3.48	0.7	2.03**	-5.43**	2.05
Race	-0.56	1.55	-0.75	-1.57**	2.31*	-2.59*
History of AF	-2.42*	4.88	-1.46	-2.22	-0.36	0.22
Diabetes	2.90*	-6.87*	3.14**	1.14	-5.25*	5.15*
Hypertension	-0.62	0.54	-0.13	-0.46	2.1	-0.49
LVEF, per %	0.12*	-0.2	0.09*	0.08	-0.17	0.1
LA Volume, per ml	0	0.03	0.01	-0.05*	0.09*	-0.11*
BMI, per kg/m2	0.1	-0.44*	0.03	0.13	-0.42*	0.29

Supplement Table 1: This table displays the four multivariable linear regression models including all variable for LA peak velocity, LA stasis, LA kinetic energy, LAA peak velocity, LAA stasis and LAA kinetic energy. Covariates which were significantly associated with each LA or LAA variable are shown. * $p < 0.05$, ** $p < 0.01$, *** $p < 0.001$

AF – atrial fibrillation, BMI – body mass index, LA – left atrium, LAA – left atrial appendage, LVEF – left ventricular ejection fraction
